# Role of COL1A1 and CD44 in Modulating JAK1/STAT3‐Mediated Autophagy for Spinal Cord Injury Recovery

**DOI:** 10.1002/kjm2.70135

**Published:** 2025-11-12

**Authors:** Chun‐Lei Li, Qian Zhang, Li Fang, Ling‐Yun Zhou

**Affiliations:** ^1^ Department of Rehabilitation Medicine The First Affiliated Hospital of Harbin Medical University Harbin City Heilongjiang China; ^2^ Department of Ocular Motility Disorder Treatment & Rehabilitation Center The First Affiliated Hospital of Harbin Medical University Harbin City Heilongjiang China

**Keywords:** autophagy, CD44, COL1A1, JAK1/STAT3 cascade, spinal cord injury

## Abstract

Spinal cord injury (SCI) is a severe trauma to the central nervous system that often leads to motor and sensory dysfunction in patients, severely affecting their quality of life. Autophagy plays a role in the pathological process of SCI, but the specific mechanism of autophagy in this case is unknown. COL1A1 and CD44, as potentially important genes in the autophagic process, may regulate the signaling pathway and thus affect the autophagic process through protein interactions. The aim of this study was to investigate the interaction between COL1A1 and CD44 and its mechanism of regulating autophagy through the JAK1/STAT3 pathway, providing new targets for SCI treatment. An SCI rat model was established, along with a PC12 cell model induced by oxygen–glucose deprivation (OGD). COL1A1 and CD44 in rat spinal cord tissues and cells were assessed using RT‐qPCR and Western blot. Motor function in rats was assessed by BBB score, and the pathological conditions of the rat spinal cord tissues and neuronal numbers were observed by HE staining and Nissl staining. COL1A1 and CD44 localization in PC12 cells was confirmed via immunofluorescence analysis, and their targeting binding was verified by Co‐IP. In the cell model, apoptosis, proliferation, and autophagy were evaluated through flow cytometry, CCK‐8, and mRFP‐GFP‐LC3 transfection, respectively. The activation of the JAK1/STAT3 cascade in spinal cord tissues and PC12 cells was assessed, along with its function in the cell model. COL1A1 and CD44 were significantly overexpressed in spinal cord tissues of SCI rats and OGD‐treated PC12 cells. COL1A1 silencing promoted functional recovery and autophagy after SCI in rats, ameliorated OGD‐induced PC12 cell injury, upregulated autophagy proteins, and increased the number of autophagosomes and autolysosomes. COL1A1 was able to bind to CD44 in a targeting fashion and regulated the JAK1/STAT3 cascade. CD44 overexpression counteracted the positive effects of COL1A1 silencing on both the functional recovery of SCI rats and OGD‐induced PC12 cell injury. COL1A1 targets and binds to CD44 to activate autophagy mediated by the JAK1/STAT3 signaling pathway, inhibiting functional recovery after SCI.

## 1 Introduction

Spinal cord injury (SCI) is a high‐risk central nervous system trauma that often causes severe motor, sensory, and autonomic dysfunction [1, 2]. With the aging of the population, the incidence of SCI has risen sharply, and prolonged suffering from SCI can cause cognitive or motor dysfunction, which seriously affects the patients' quality of life, but the mechanism has not yet been elucidated [3, 4]. Patients with SCI commonly face immense physical and psychological challenges, and the financial burden of treatment and care weighs heavily on them, their families, and society. SCI has emerged as a significant global health challenge that needs addressing, yet a fully effective treatment remains elusive. For this reason, delving into the molecular mechanisms of SCI pathophysiology is important.

The pathophysiology of SCI can be succinctly categorized into two phases: primary injury and secondary injury [5, 6, 7]. SCI primary injury is caused by mechanical trauma leading to nerve compression, axonal rupture, and damage to blood vessels and cell membranes [8], while secondary injury arises from inflammation, vascular changes, edema, necrosis, apoptosis, and autophagy [9]. The central nervous system relies on autophagy as a key catabolic process to ensure a healthy and stable homeostasis [10]. Research indicates that autophagy can lessen neuronal damage and enhance motor recovery by preventing neuronal apoptosis in SCI rats [8]. Autophagy activation is key to protecting neuronal cells because of the accumulation of damaged organelles and proteins during SCI [11]. Through the breakdown of damaged organelles, mitochondria, nucleic acids, and proteins, autophagy is intricately involved in managing immune and inflammatory responses, forming autophagosomes that direct cytoplasmic contents to the lysosome. Therefore, autophagy is often used as a therapeutic phenotypic target for SCI [10, 12, 13]. Autophagy is controlled by several cascades, with the JAK/STAT pathway being a key player [14, 15]. Additionally, the JAK1/STAT3 signaling pathway is involved in SCI. The pan‐JAK inhibitor tofacitinib (TOF) can modulate glial cell polarization through the JAK/STAT signaling pathway, thereby promoting functional recovery following SCI [16].

The gene known as COL1A1 encodes the pro‐alpha 1 chain of type I collagen, featuring a triple helix structure composed of two alpha 1 chains and a single alpha 2 chain [17]. High COL1A1 expression is associated with the progression of various cancers [18, 19, 20]. In addition, COL1A1 is involved in the regulation of cellular autophagy [21]. COL1A1 is persistently elevated following SCI and could be implicated in stromal remodeling, fibrosis, and the formation of scars [22]. So far, there has been no investigation into the role of COL1A1 in the SCI process. Hence, this study explored the regulation of cellular autophagy by COL1A1 in relation to functional recovery after SCI.

## 2 Materials and Methods

### 2.1 Experimental Animals

Sprague–Dawley rats (*n* = 48), males weighing between 250 and 300 g and aged 6–8 weeks, were sourced from Cyagen Biosciences located in Suzhou, China. All SD rats were housed in 12 cages of 4 rats each under standard conditions. They were maintained under standard conditions, with temperatures between 21°C and 25°C, a 12‐h light/dark cycle, and humidity levels of 50%–60%, and had free access to food and water. Animals were grouped by simple randomization (rats were randomly assigned to groups by generating random numbers, ranking them, and assigning them to predetermined groups to ensure unbiased allocation in experiments), and their group identity was unknown to the researchers who performed the functional assessments and image analyses.

### 2.2 Animal Model

The sample size was calculated based on the sample size of 6–12 rats per group, and in this study, under the premise of ensuring the reliability of the experimental data and based on the 3R principle of animal experiments, the sample size was determined to be 48 rats, with 8 rats in each group. Rats were randomly divided into sham group, SCI group, sh‐NC group (SCI‐treated and injected with silenced negative control lentivirus), sh‐COL1A1 group (SCI‐treated and injected with silenced COL1A1 lentivirus), sh‐COL1A1 + oe‐NC group (SCI‐treated and injected with silenced COL1A1 and overexpressed negative control lentivirus), sh‐COL1A1 + oe‐CD44 group (SCI‐treated and injected with silenced COL1A1 and overexpressed CD44 lentivirus), with eight rats in each group. All surgical procedures were conducted using 1% pentobarbital sodium anesthesia at a dosage of 20 mg/kg (Sigma‐Aldrich). Sham group rats underwent sham surgery for laminectomy, during which the T10 spinal cord was exposed, and the muscle, fascia, and skin were stitched up one after the other. The remaining rats were set up as an SCI model using the modified Allen method. After deep anesthesia and skin preparation, the surgical region was sterilized with iodine vapor. The T9 to T10 processes were pinpointed using bony landmarks, and an incision was made in the skin over these landmarks. The muscle tissue was bluntly separated, exposing the T10 lamina and spinous process. The lamina was resected to expose the spinal cord tissue. Using the NYU spinal cord striker, a 10 g weight was dropped from a height of 2.5 cm above the dorsal median part of the spinal cord (vertical distance), resulting in SCI. Involuntary spasms in the hind limbs and tail twisting in the rats suggested that the modeling was successful. The muscle and skin tissues were stitched, and 200,000 units per day of penicillin were administered subcutaneously for 3 days after surgery to prevent infection. Additionally, the bladder was massaged twice daily, in the morning and evening, to aid urination until voluntary control returned. Lentiviral therapy was performed 3 days after laminectomy (days 0, 1, and 2). sh‐NC, sh‐COL1A1, oe‐NC, and oe‐CD44 lentiviruses (50 μL/day, 100 nmoL/mL; Reebok Biologics, Guangzhou, China). Intrathecal injection was performed via lumbar puncture at the L5‐6 interspinous space for 15 min per day.

### 2.3 Basso, Beattie, and Bresnahan (BBB) Scale

In SCI rats, motor function was measured using the BBB scale, which spans from 0 to 21. A score of 0 denotes complete paralysis with no visible hind limb movement, while a score of 21 indicates complete activity. The rats were placed in an open field for 5 min each day (AM) on days 1, 3, 5, 7, 14, 21, and 28 after surgery. Each rat underwent a 4‐min assessment, with three skilled researchers documenting the hind limb movement scores.

### 2.4 HE Staining

An overdose of sodium pentobarbital was given intraperitoneally to euthanize the rats on Day 28 after surgery. After opening the spine, about 3 cm of spinal cord tissue was taken from the center of the SCI, and the spinal cord tissue was fixed with 4% paraformaldehyde (Beyotime, P0099) at 25°C for 48 h. The tissue was subsequently dehydrated in ethanol gradients and permeabilized with xylene. Next, the tissues were embedded in paraffin, cut into 4 μm thick sections, stained with hematoxylin for 2 min at 25°C, stained with eosin for 1 min, and sealed with neutral gum. The morphological changes of the spinal cord tissue were photographed and observed under a BX51 light microscope (Olympus, Japan). Morphological changes of spinal cord tissues were photographed and observed under a BX51 light microscope (Olympus, Japan) in five randomly selected field views.

### 2.5 Nissl Staining

The previously mentioned 4 μm slices were immersed in an equal mix of absolute ethanol and chloroform, allowing them to permeabilize throughout the night. Subsequently, the samples underwent treatment using 100% absolute ethanol, 95% ethanol, and distilled water. The samples underwent pre‐heating with 0.1% cresyl violet at 37°C for 10 min, were differentiated using 95% ethanol for 5 min, and subsequently immersed in ethanol and xylene. Ultimately, the sections were prepared for microscopic examination. Five visual fields of the anterior horn of the spinal cord were observed under a 400‐fold microscope, and the number of Nissl‐stained positive cells in the visual field was recorded.

### 2.6 Cell Culture and Transfection

The rat PC12 (ATCC, USA) was cultured in Dulbecco's Modified Eagle's Medium (Sigma‐Aldrich) containing 10% fetal bovine serum (Hyclone, UT, USA) at 37°C, 5% CO_2_. The cells used in the experiment were in the logarithmic growth phase. Following this, the cells were subjected to oxygen–glucose deprivation/reoxygenation (OGD/R) as previously detailed [23]. The procedure involved exposing PC12 cells to OGD for 4 h, after which they received a 24‐h treatment with R. COL1A1 si‐RNA, control si‐RNA (si‐NC), CD44 overexpression vector (oe‐CD44), and empty vector (oe‐NC) were purchased from Genepharma (China). PC12 cells subjected to OGD/R were inoculated into 6‐well plates and transiently transfected with lipofectamine 3000 (Invitrogen, USA). The cells were harvested 48 h afterward.

### 2.7 CCK‐8 Assay

PC12 cell proliferation was examined using the CCK‐8 kit (Beo Tianmei, China). PC12 cells were located in 96‐well plates with 1 × 10^4^ cells per well and incubated for 24 h. Post OGD/R and transfection, 10 μL of CCK‐8 reagent was combined with PC12 cells for an hour at 37°C. Subsequently, a microplate reader (Biotek, VT, USA) was utilized to measure the optical density at 450 nm.

### 2.8 Flow Cytometry

PC12 apoptosis was detected by the Annexin V‐FITC Apoptosis Detection Kit (BD Biosciences, CA, USA). After being resuspended in binding buffer, the cell density was adjusted and inoculated at 3 × 10^5^ cells per well in a 6‐well cell culture plate, with 3 replicate wells set up for each group. Then, 500 μL of the PC12 cell suspension was incubated with 5 μL Annexin V‐FITC and 10 μL PI for 10 min. Finally, the apoptosis rate was assessed by flow cytometry (BD Biosciences).

### 2.9 Autophagy Flux Assay

PC12 cells underwent co‐transfection using the mRFP‐GFP‐LC3 plasmid (Hanbio, Shanghai, China) alongside si‐COL1A1 or oe‐CD44. The density of PC12 cells was adjusted to 1 × 10^5^ cells/mL. Then, 1 mL of the cell suspension was seeded in a cell culture dish, and after the cells were adhered to the wall, the medium was removed and replaced with serum‐free medium, and 20 μL of adenovirus mRFP‐GFP‐LC3 was added. Post‐transfection, the cells underwent a culture period of 48 h. Following this, the cells were fixed using 4% paraformaldehyde for a quarter‐hour and were subjected to 1 × Hoechst 33,342 nuclear staining for 30 min. The fluorescence of mRFP and GFP was captured through a confocal laser scanning microscope (Leica). Autophagosomes appear as yellow dots while autophagic lysosomes are represented by red dots. ImageJ software was employed to measure the quantity of yellow and red dots. The flow of autophagy was assessed by determining the average count of autophagosomes and autolysosomes.

### 2.10 Immunofluorescence Analysis

Cells were exposed to 4% paraformaldehyde for 20 min at 4°C, blocked with a mixture of 5% skimmed milk and 0.1% Triton X‐100 for 1 h at room temperature, and incubated with primary antibody overnight at 4°C. The subsequent day, cells were exposed to Alexa Fluor‐labeled secondary antibody for 60 min, followed by a 30‐min incubation with 4′‐6‐diamidino‐2‐phenylindole, and then observed with a confocal microscope (Nikon, ECLIPSE Ti2).

### 2.11 Co‐IP

Co‐IP assay was performed using the Pierce Co‐IP kit (Thermo Fisher Scientific, USA). PC12 cells were lysed on ice using IP lysis buffer, treated with agarose resin for 1 h at 4°C, and incubated with COL1A1, CD44, and IgG antibodies overnight at 4°C. Captured antigens were eluted and subjected to SDS‐PAGE analysis, with protein levels being measured using western blot.

### 2.12 RT‐qPCR

Total RNA extraction from cells and tissues was performed utilizing TRIzolTM Reagent (Thermo Fisher Scientific). Using Roche's Transcriptor Reverse Transcriptase, cDNA was created from 1 μg of total RNA. The PCR process utilized SYBR Green PCR Master Mix (Applied Biosystems; Thermo Fisher Scientific) on a 7500HT Fast Real‐Time PCR System (Applied Biosystems; Thermo Fisher Scientific). Table 1 contains the list of primers. Gene expression was standardized to GAPDH and determined using the 2^−ΔΔCt^ method.

**TABLE 1:** Primer sequences.

Genes	Forward	Reverse
*COL1A1*	GGAGAGAGCATGACCGATGG	TTCGATGACTGTCTTGCCCC
*CD44*	CTCAAAAAGCCATGCAACAGC	CTCCGTACCAGGCATCTTCG
*GAPDH*	GTCGGTGTGAACGGATTTG	TCCCATTCTCAGCCTTGAC

*Note: COL1A1*, Collagen type I alpha 1; *GAPDH*, glyceraldehyde 3‐phosphate dehydrogenase.

### 2.13 Western Blot

Total proteins from spinal cord tissues or cells were extracted by RIPA buffer (Beyotime). Nuclear and cytoplasmic proteins were isolated using the Nucleus and Cytoplasmic Protein Extraction Kit (Beyotime). All isolated proteins were quantified by the BCA protein assay kit (Boster, Wuhan, China). Protein samples totaling 40 μg were separated using 10% SDS‐PAGE and then transferred to a PVDF membrane. After sealing with 5% skimmed milk, the membrane was incubated with the primary antibody at 4°C overnight and the secondary antibody goat anti‐rabbit IgG H&L (ab6721, Abcam) for 1 h at 25°C. Finally, the membrane was visualized using ECL detection reagents (Thermo Fisher Scientific) and analyzed using ImageJ software. Antibodies used included Beclin‐1 (ab207612, Abcam), LC3II/LC3I (ab192890, Abcam), COL1A1 (#PB0981, BOSTER), CD44 (ab51037, Abcam), GAPDH (ab9485, Abcam), JAK1 (ab133666, Abcam), STAT3 (ab68153, Abcam), p‐JAK1 (E‐AB‐20913, ELabscience), and p‐STAT3 (ab32143, Abcam).

### 2.14 Statistical Analysis

Statistical analysis was performed using GraphPad Prism 8.0 (GraphPad Software Inc., La Jolla, USA). Parametric data are expressed as mean ± standard deviation (SD), and nonparametric data are expressed as median ± interquartile range. The Shapiro–Wilk test was used to evaluate the normality of the data, and the F‐test was used to test the homogeneity of variance. One‐way ANOVA or paired‐sample t‐test was used for intergroup comparisons if the data met normal distribution and variance was homogeneous, and the LSD‐t method was used for multiple comparisons; the Welch‐*t* test was used if the variance was not homogeneous. If the data did not satisfy normal distribution, they were analyzed using the Mann–Whitney *U* nonparametric test. Behavioral data from multiple time points were analyzed using two‐factor repeated‐measures ANOVA followed by Tukey's multiple comparison test. Two‐sided tests were performed, and *p* < 0.05 was considered a statistically significant difference.

## 3 Results

### 3.1 COL1A1 Silencing Promotes Functional Recovery and Autophagy After SCI in Rats

**FIGURE 1:**
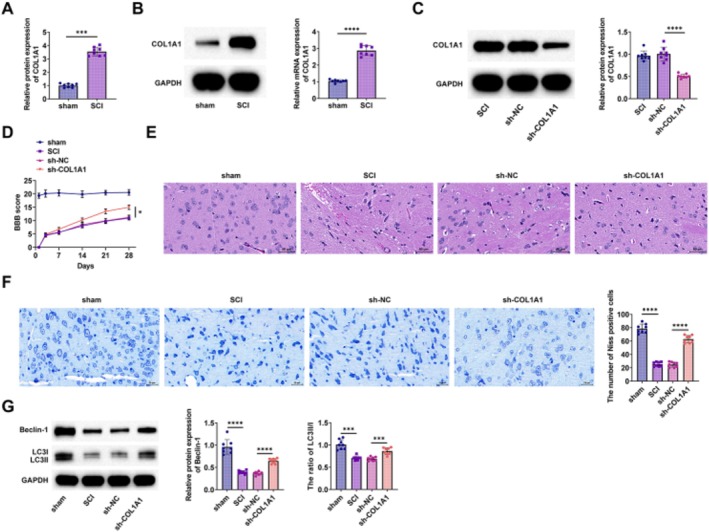
COL1A1 silencing promotes functional recovery and autophagy after SCI in rats. (A) RT‐qPCR detected the expression of *COL1A1* in the spinal cord tissues of rats in each group; (B) Western blot detected the expression of COL1A1 in the spinal cord tissues of rats in each group; (C) Western blot detected the expression of COL1A1 in the spinal cord tissues of rats after knocking down COL1A1; (D) BBB scores of rats in each group; (E) HE staining observed the pathological and morphological damage of spinal cord tissues. Scale bar = 50 μm. (F) Nissl staining observed the number of motor neurons in rat spinal cord, scale bar = 25 μm; (G) Western blot detected Beclin‐1 and LC3II/LC3I in the spinal cord tissues of rats in each group. *p < 0.05, ***p < 0.001, ****p < 0.0001.

While COL1A1 is known to be consistently elevated in SCI [24], its mechanism of action in SCI is not well defined. COL1A1 in the spinal cord tissues of rats was analyzed using RT‐qPCR and Western blot, showing a marked increase in SCI rats (Figure 1A,B). Next, the effect of COL1A1 on the functional recovery of SCI rats was determined by knocking down COL1A1. The sh‐COL1A1 knockdown vector was administered to SCI rats, and its knockdown efficiency was confirmed using Western blot analysis (Figure 1C). The recovery of motor function in rats after SCI surgery was measured with the BBB scale, showing that COL1A1 knockdown aided in motor function recovery (Figure 1D). HE staining demonstrated that the spinal cord ventral horn of SCI rats, as shown by HE staining, exhibited structural disorganization with intracellular and intercellular vacuoles and cavities, which was reduced after COL1A1 knockdown (Figure 1E). Nissl staining demonstrated a significant reduction in the number of retained spinal ventral horn motor neurons after SCI compared with the sham group. However, the number of retained motor neurons increased after COL1A1 silencing (Figure 1F). Apoptotic proteins Beclin‐1 and LC3II/LC3I in rat spinal cord tissues were detected using Western blot. The data revealed that knocking down COL1A1 resulted in a notable increase in Beclin‐1 and LC3II/LC3I expression (Figure 1G).

### 3.2 COL1A1 Silencing Ameliorates OGD‐Induced PC12 Cell Injury and Enhances Autophagy

**FIGURE 2:**
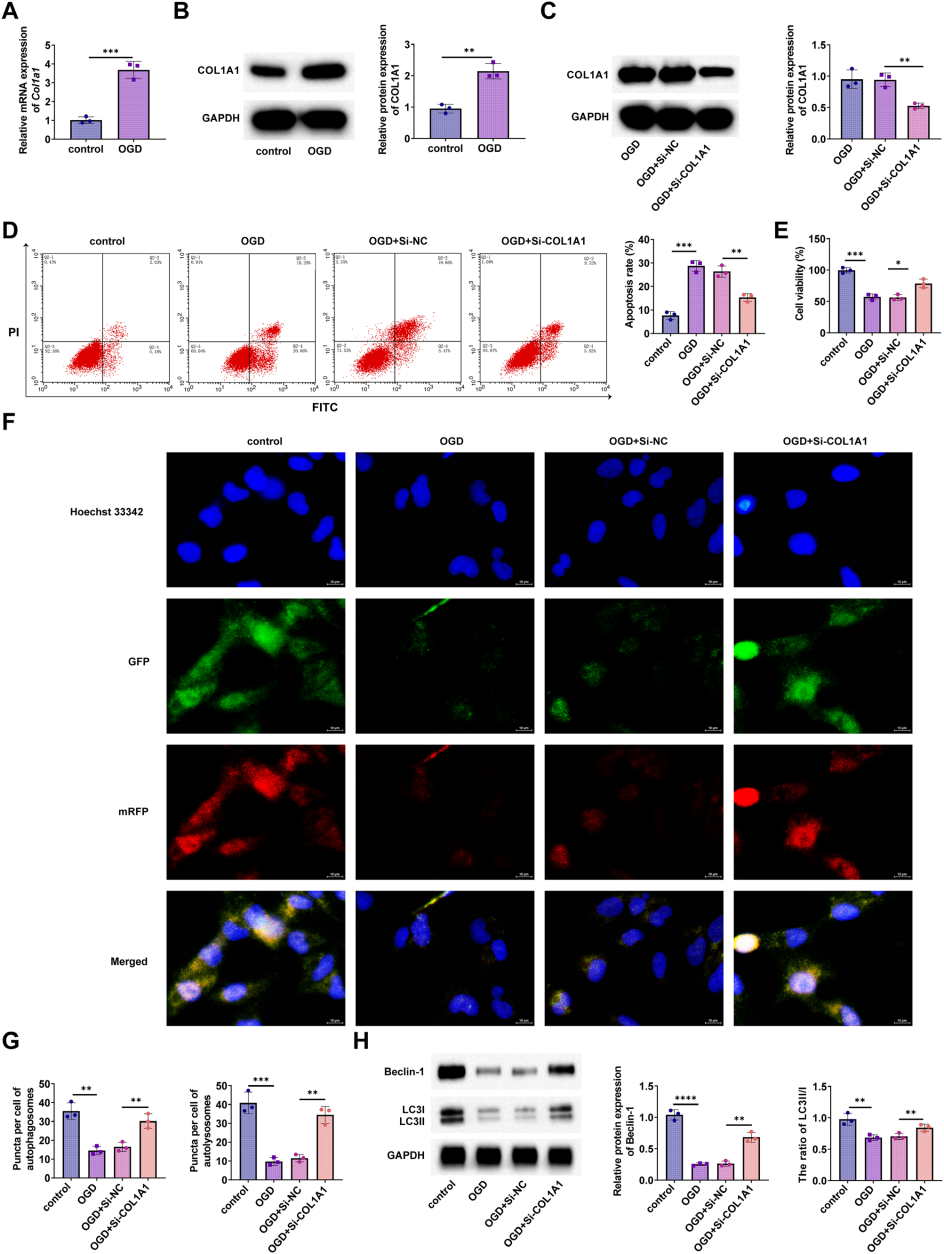
COL1A1 silencing ameliorates OGD‐induced PC12 cell damage and enhances autophagy. (A, B) RT‐qPCR and Western blot detected *COL1A1* mRNA and COL1A1 protein expression in each group of cells; (C) Western blot verified the knockdown effect of COL1A1 in PC12 cells; (D) Flow cytometry detected apoptosis; (E) CCK‐8 detected cell proliferation; (F, G) Fluorescence images of PC12 after cotransfection of mRFP‐GFP‐LC3 plasmid and si‐NC or si‐COL1A1. Scale bar = 50 μm. (H) Western blot detected Beclin‐1 and LC3II/LC3I expression in each group of cells. *p < 0.05, ***p < 0.001, ****p < 0.0001.

COL1A1 expression was measured in each group of PC12 cells to study its role in the cell model of SCI induced by OGD. RT‐qPCR and Western blot results demonstrated that COL1A1 was significantly upregulated after being induced by OGD (Figure 2A,B). Transfection of si‐COL1A1 into OGD‐induced PC12 cells was performed to knock down COL1A1 expression. Western blot verified the knockdown effect (Figure 2C). COL1A1 silencing exerted an anti‐apoptotic effect on PC12 cells by flow cytometry (Figure 2D). CCK‐8 assay demonstrated that COL1A1 silencing enhanced the cell viability of PC12 (Figure 2E). The role of COL1A1 in regulating cellular autophagy was explored using the mRFP‐GFP‐LC3 plasmid, which serves as a reporter gene for autophagy flux. COL1A1 silencing increased the number of autophagosomes (yellow dots) and autophagolysosomes (red dots) in PC12 cells (Figure 2F,G). Western blot results demonstrated that OGD inhibited Beclin‐1 and LC3II/LC3I expression, whereas COL1A1 silencing increased Beclin‐1 and LC3II/LC3I protein expression (Figure 2H).

### 3.3 COL1A1 Binds to CD44

**FIGURE 3:**
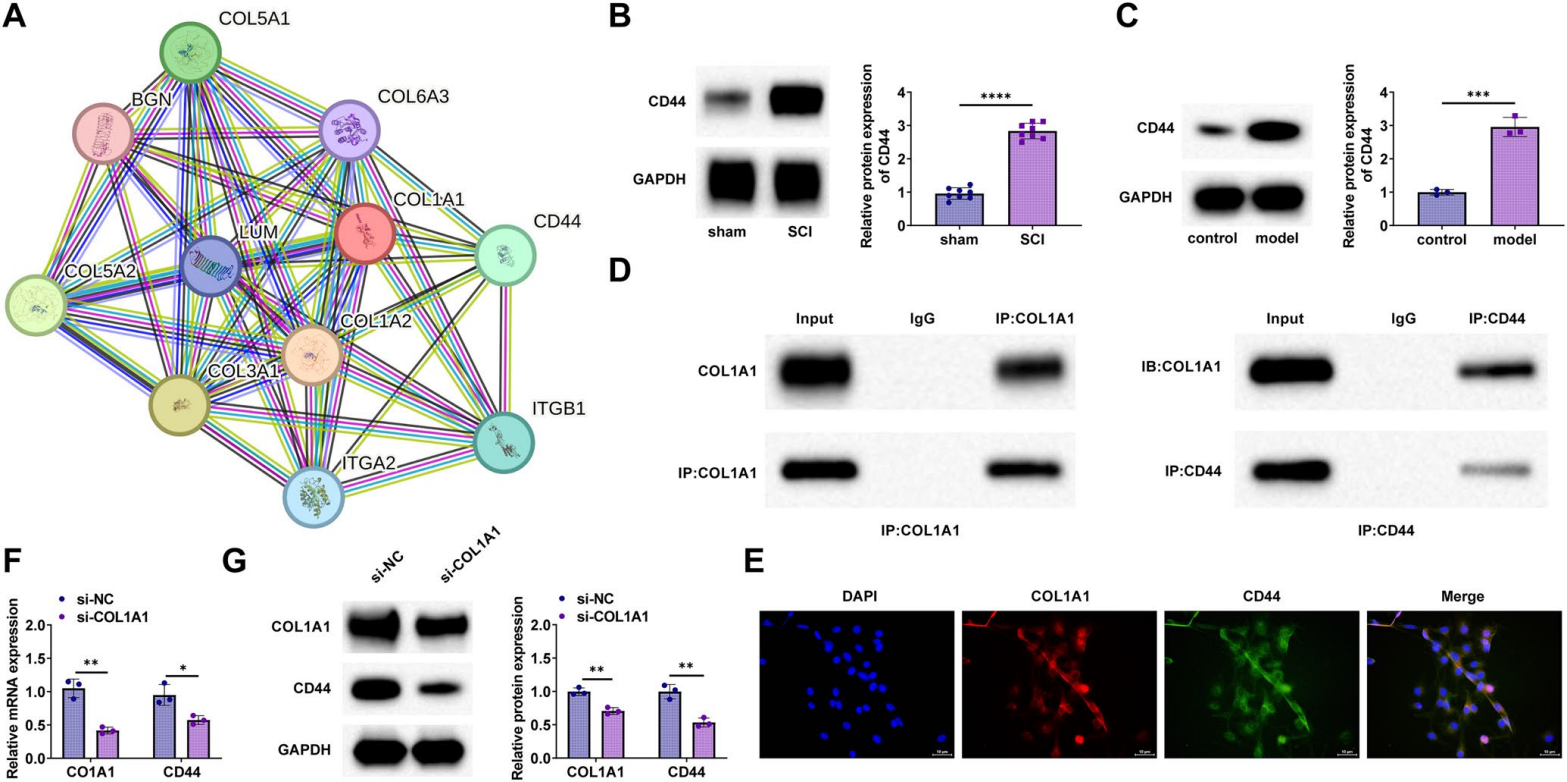
COL1A1 binds to CD44 targeting. (A) STRING database demonstrated the interactions between COL1A1 and CD44; (B, C) Western blot detected CD44 expression in SCI spinal cord tissues and OGD‐treated PC12 cells; (D) Co‐IP validated targeted binding of COL1A1 to CD44; (E) Immunofluorescence detected cellular co‐localization of COL1A1 with CD44; (F) RT‐qPCR detected the mRNA expression trend of *COL1A1* and *CD44* in PC12 cells after knocking down COL1A1; (G) Western blot detected the protein expression trend of COL1A1 and CD44 in PC12 cells after knocking down COL1A1. *p < 0.05, ***p < 0.001, ****p < 0.0001.

Based on the STRING database, the COL1A1 protein network, shown in Figure 3A, predicted an interaction between COL1A1 and CD44. We detected the expression of CD44 in SCI spinal cord tissues, OGD‐PC12, by Western blot. CD44 expression was upregulated in SCI spinal cord tissues and OGD‐treated PC12 cells (Figure 3B,C). To verify the interaction between COL1A1 and CD44, a CO‐IP assay was performed in PC12 cells stably transfected with COL1A1 (Flag‐tagged). The results, as shown in Figure 3D, demonstrated that COL1A1 directly interacted with CD44. Immunofluorescence further confirmed that COL1A1 co‐localized with CD44 in the cytoplasm of PC12 (Figure 3E). In the COL1A1‐knockdown PC12 cell line, the expression relationship between *COL1A1* and *CD44* was assessed at the mRNA (Figure 3F) and protein (Figure 3G) levels using PCR and Western blot, indicating a positive correlation.

### 3.4 CD44 Overexpression Negates the Functional Improvements From COL1A1 Knockdown in SCI Rats

**FIGURE 4:**
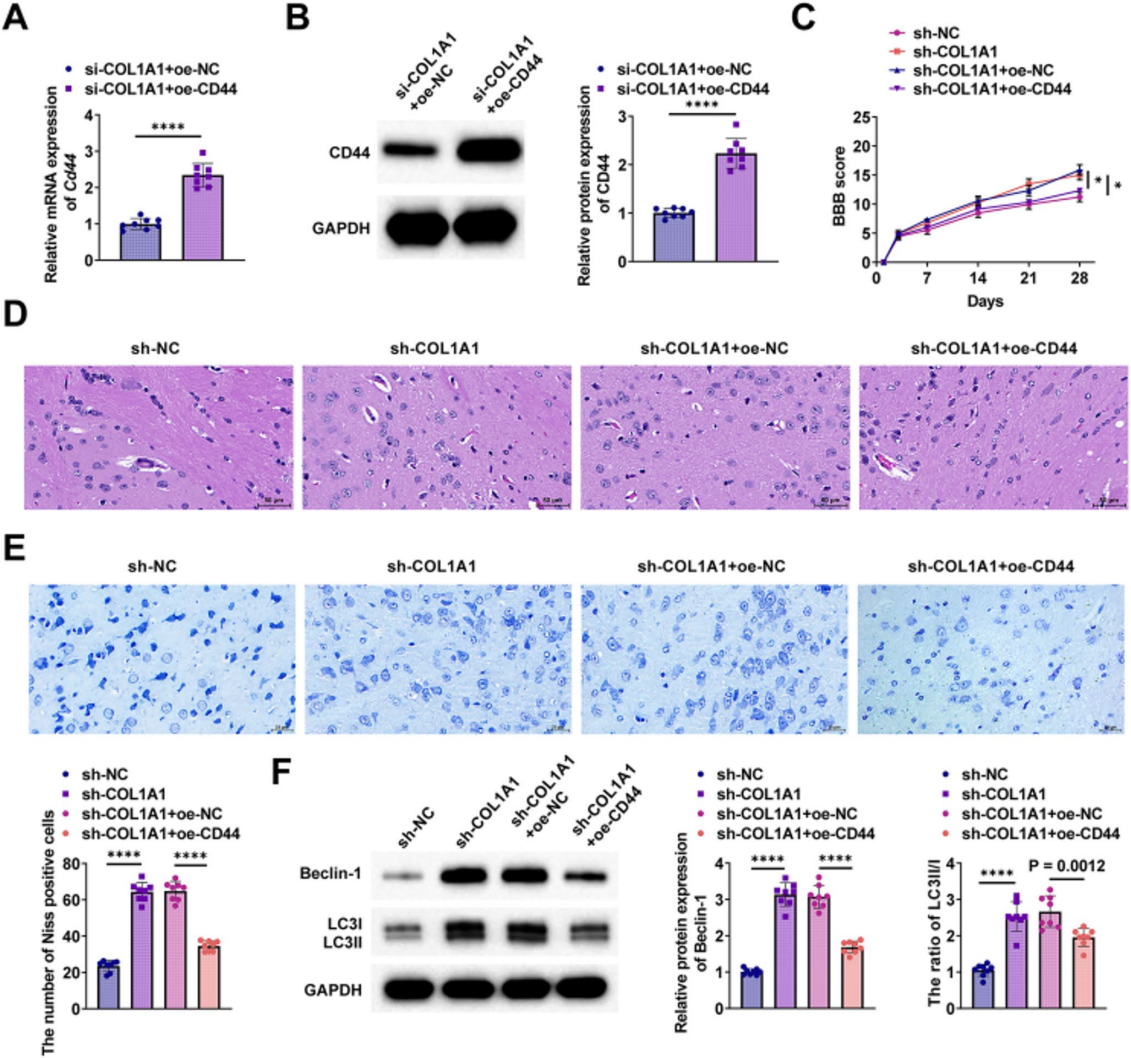
CD44 overexpression negates the functional improvements from COL1A1 knockdown in SCI rats. (A, B) RT‐qPCR and Western blot verified the transfection efficiency of overexpression vector oe‐CD44; (C) BBB score analyzed the neurological function of rats; (D) HE staining observed the pathological condition of rat spinal cord tissues, scale bar = 50 μm; (E) Nissl staining observed the number of motor neurons in spinal cord tissues, scale bar = 25 μm; (F): Western blot detected the expression of Beclin‐1 and LC3II/LC3I in the spinal cord of rats. *p < 0.05, ****p < 0.0001.

Functional exploration was performed in SCI rats by injecting sh‐NC, sh‐COL1A1, sh‐COL1A1 + oe‐NC, and sh‐COL1A1 + oe‐CD44 into rats, respectively. The transfection efficiency of the CD44 overexpression vector oe‐CD44 was first verified using RT‐qPCR and Western blot (Figure 4A,B). Motor function recovery in rats following SCI surgery was evaluated using the BBB scale, showing that the effect of COL1A1 knockdown on recovery was negated by CD44 overexpression (Figure 4C). HE staining indicated that CD44 overexpression counteracted the impact of COL1A1 silencing, which led to a decrease in intracellular and intercellular vacuoles and lumens, as well as disorganized cellular structures in rat spinal cord tissues (Figure 4D). The increase in motor neuron numbers due to COL1A1 silencing was reversed by CD44 overexpression, as shown by Nissl staining (Figure 4E). According to Western blot findings, CD44 overexpression counteracted the increase in Beclin‐1 and LC3II/LC3I induced by COL1A1 silencing in the spinal cord tissues of rats (Figure 4F).

### 3.5 CD44 Overexpression Negates the Benefits of COL1A1 Knockdown on OGD‐Induced Injury in PC12 Cells

**FIGURE 5:**
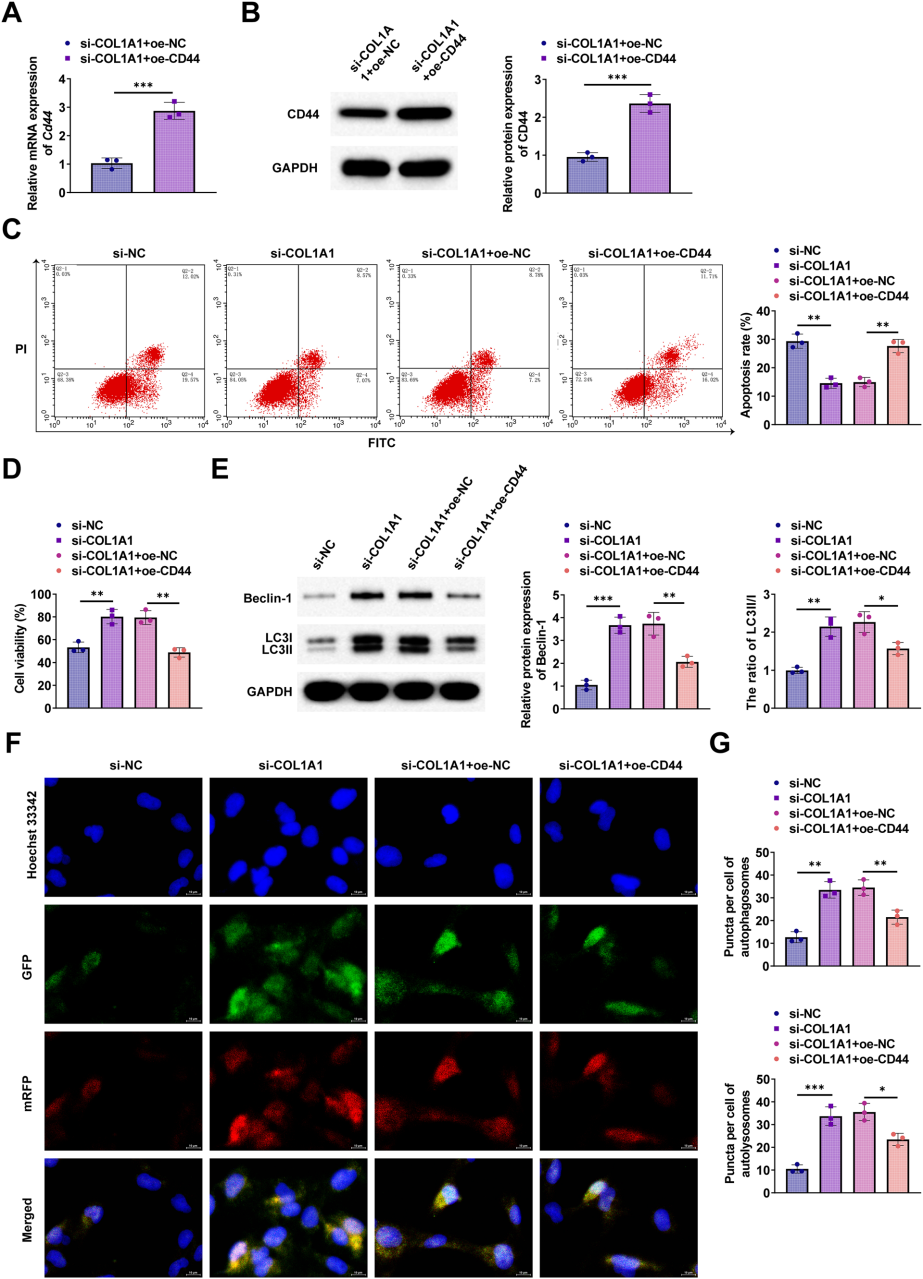
CD44 overexpression reverses the ameliorative effect of COL1A1 silencing on OGD‐induced PC12 cell injury. (A, B) RT‐qPCR and Western blot verified the transfection efficiency; (C) Flow cytometry detected apoptosis; (D) CCK‐8 detected cell proliferation; (E) Western blot detected the expression of Beclin‐1 and LC3II/LC3I; (F, G) PC12 cells were co‐transfected with mRFP‐GFP‐LC3 plasmid to detect cellular autophagic flux. *p < 0.05, ***p < 0.001.

To further investigate the mechanism of COL1A1 and CD44 in SCI, an overexpression plasmid of CD44 was transfected into PC12 cells to upregulate CD44 expression. RT‐qPCR and Western blot verified the transfection efficiency (Figure 5A,B). Flow cytometry revealed that COL1A1 silencing exerted an anti‐apoptotic effect on PC12 cells, while knockdown of CD44 was able to reverse the effect (Figure 5C). CCK‐8 assay demonstrated that the enhancement of cell proliferation of PC12 by COL1A1 silencing was negated by CD44 overexpression (Figure 5D). Western blot assay demonstrated that the upregulation of Beclin‐1 and LC3II/LC3I by COL1A1 silencing was reversed by CD44 overexpression (Figure 5E). Autophagy flux analysis demonstrated that CD44 overexpression counteracted the increase in the number of autophagosomes and autophagolysosomes by COL1A1 silencing (Figure 5F,G).

### 3.6 COL1A1 Regulates the JAK1/STAT3 Cascade Through CD44

**FIGURE 6:**
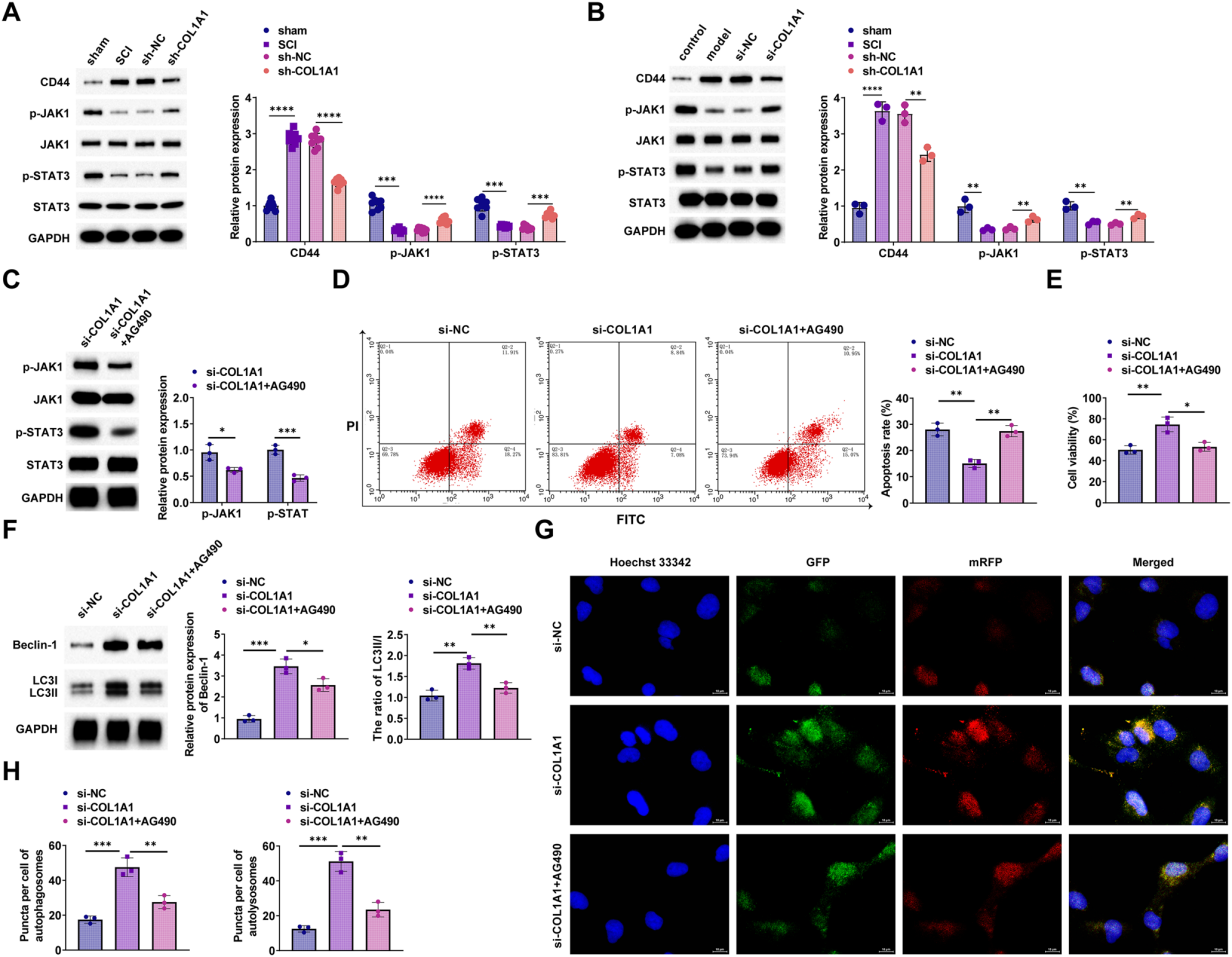
COL1A1 regulates JAK1/STAT3 cascade through CD44. (A) Western blot detected CD44 and JAK1/STAT3 cascade protein expression in spinal cord tissues of SCI rats with COL1A1 silencing; (B) Western blot detected CD44 and JAK1/STAT3 cascade protein expression in PC12 cells with COL1A1 silencing; (C) Western blot detected the effect of JAK1/STAT3 cascade inhibitor AG‐490 on JAK1/STAT3 cascade proteins in PC12 cells; (D) Flow cytometry detected the effect of AG‐490 on apoptosis; (E) CCK‐8 detected the effect of AG‐490 on cell proliferation; (F) Western blot detected the effect of AG‐490 on autophagy proteins in PC12 cells; (G, H): Effect of AG‐490 on cellular autophagic flux. *p < 0.05, ***p < 0.001, ****p < 0.0001.

The involvement of the JAK1/STAT3 cascade in SCI is significant [25], but its role in regulating COL1A1 and CD44 remains uncertain. This study proposes that COL1A1/CD44 is involved in SCI via the regulation of this pathway. Western blot results demonstrated that in the spinal cord tissues of rats with SCI that knocked down COL1A1, CD44 expression was downregulated, and the JAK1/STAT3 cascade was activated, accompanied by upregulated p‐JAK1 and p‐STAT3 (Figure 6A). It was also observed in PC12 cells that CD44 was significantly downregulated and p‐JAK1 and p‐STAT3 were upregulated in cells after knocking down COL1A1 (Figure 6B).

To understand the role of the JAK1/STAT3 cascade in COL1A1, CD44‐mediated SCI injury, a selective JAK1/STAT3 cascade inhibitor, AG‐490, was added in vitro to block the phosphorylation of JAK1 and STAT3. Western blot indicated that AG‐490 could effectively inhibit the activity of the JAK1/STAT3 cascade and suppress p‐JAK1 and p‐STAT3 expression (Figure 6C). The suppression of PC12 cell apoptosis by COL1A1 knockdown was counteracted by AG‐490, as demonstrated by flow cytometry (Figure 6D). Also, the CCK‐8 assay indicated that AG‐490 counteracted the increase in PC12 cell proliferation by silencing COL1A1 (Figure 6E). Western blot analysis indicated that AG‐490 inhibited the enhancing effect of COL1A1 silencing on autophagy in PC12 cells (Figure 6F). Moreover, AG‐490 reversed the promoting effect of knocking down COL1A1 on cellular autophagic flux (Figure 6G,H).

## 4 Discussion

SCI is highly disabling as it causes severe leg dysfunction or permanent paralysis in patients and rapidly triggers secondary damage that leads to neuronal injury [26]. The process of autophagy collaborates with the ubiquitin‐proteasome system to degrade damaged organelles; however, excessive autophagy can lead to the breakdown of cellular components and result in cell death. Autophagy has been demonstrated to have a significant role in the context of SCI [24]. On one side, boosting autophagy supports motor recovery in SCI, but on the flip side, when autophagy is compromised post‐SCI, it intensifies neuroinflammation and motor problems in mice [10]. Although there is much debate about autophagy's role in SCI, more evidence is pointing towards its beneficial effects in SCI [27, 28].

The COL1A1 gene encodes the α1 chain of type I collagen, which is a major component of the extracellular matrix [29]. Type I collagen, prevalent in tissues like skin, bone, tendons, and ligaments, offers essential strength and structural support. The ongoing upregulation of COL1A1 in SCI signifies the predominance of inflammation and scarring following SCI, which fosters pathological matrix remodeling and protein hydrolysis [22]. The study by Shu et al. revealed notably increased COL1A1 levels in the blood of SCI patients, underlining its association with neuroinflammation post‐SCI [30]. In this study, COL1A1 expression was abnormally elevated in the spinal cord tissues of SCI rats, and COL1A1 silencing promoted the recovery of motor function, improved the pathological morphology of spinal cord tissues, increased the number of motor neurons, and promoted cellular autophagy in spinal cord tissue. High expression of COL1A1 was also observed in OGD‐treated PC12 cells. COL1A1 silencing could inhibit apoptosis and enhance cell proliferation. Metformin treatment in traumatic SCI rats not only improves functional recovery but also reduces apoptosis, enhances autophagosome formation, and increases the expression of autophagy biomarkers such as Beclin‐1 and LC3B II, while decreasing accumulation of autophagy substrate protein p62 and ubiquitylated proteins. This suggests a stimulation of autophagic flux [31]. Similarly, this study observed that COL1A1 silencing increased the number of autophagosomes and autolysosomes in PC12 cells. COL1A1 silencing induced the upregulation of Beclin‐1 and LC3II/LC3I in spinal cord tissues and OGD‐treated PC12 cells. Facilitating autophagy in traumatic SCI enables the degradation and recycling of intracellular contents, which supports neuronal survival in conditions where trophic factors are deficient. As a result, promoting autophagy in traumatic SCI is regarded as a promising approach for neuroprotection [32, 33].

As an adhesion molecule, CD44 is involved in cell–cell and cell–matrix interactions, participating in various physiological and pathological processes [34]. The gene CD44 is central to SCI and potentially crucial for the autophagy process that follows [35]. This study, like previous ones, found an increase in CD44 expression in spinal cord tissues of SCI rats and PC12 cells treated with OGD, and it also demonstrated that COL1A1's targeted binding to CD44 played a role in the SCI autophagy process. CD44 overexpression reversed the ameliorative effect of COL1A1 silencing on SCI rats and OGD‐treated PC12 cells.

Among the most significant pathways for cytokine signaling from the cell surface to the nucleus is the JAK/STAT pathway [36]. Recent research indicates that triggering the JAK1/STAT3 cascade enhances mitochondrial function and reduces lipid peroxidation in vascular endothelial cells following traumatic SCI [37]. JAK1/STAT3 is often used as a correlative pathway of inflammation involved in SCI [38, 39]. In SCI, autophagy and inflammation are inextricably linked, and genetic inhibition of autophagy induces a proinflammatory response to injury that adversely affects tissue damage and recovery of motor function after SCI [10]. Our study found that the JAK1/STAT3 cascade had not been activated in SCI, and COL1A1 silencing activated the JAK1/STAT3 cascade and promoted cellular autophagy. This result suggests that the JAK1/STAT3 cascade is involved in the autophagic response of neurons after SCI, and the activation of this pathway promotes autophagy, and the inhibitory effect of high COL1A1 on autophagy in rat spinal cord neurons after SCI may be related to its inhibition of the activation of the JAK1/STAT3 cascade.

Although this study revealed the mechanism by which COL1A1 affects the functional recovery of SCI by activating autophagy through targeting CD44 and activating the JAK1/STAT3 signaling pathway, there are some limitations. The research primarily utilized the SD rat SCI model and the PC12 cell OGD/R model, acknowledging that the pathological complexity in the animal model may not fully replicate that of human SCI. The cellular model in vitro cannot fully simulate the microenvironment in vivo. The experiments focused on the COL1A1‐CD44‐JAK1/STAT3‐autophagy axis and did not delve into other potential signaling pathways or molecular interactions on SCI. The functional assessment mainly relied on BBB scoring and pathological staining and lacked a more comprehensive validation of behavioral or electrophysiological indicators. In addition, the application of the JAK1/STAT3 pathway inhibitor AG‐490 supported the pathway association but did not exclude other nonspecific effects, and the in vivo rescue experiments of overexpression of CD44 did not elucidate the specific molecular regulation details, which need to be combined with more clinical samples and multidimensional experimental models to further validate the mechanism generality.

In summary, COL1A1 silencing improves functional recovery after SCI by targeting binding to CD44 to activate autophagy mediated by the JAK1/STAT3 cascade. Not only does this study search for SCI therapeutic targets, but it also contributes additional information on the autophagy mechanism in SCI. However, detailed studies on other regulatory mechanisms of COL1A1 in SCI are still needed.

## Ethics Statement

All animal experiments were complied with the ARRIVE guidelines and performed in accordance with the National Institutes of Health Guide for the Care and Use of Laboratory Animals. The experiments were approved by the Institutional Animal Care and Use Committee of The First Affiliated Hospital of Harbin Medical University (No. HB20221147‐5).

## Conflicts of Interest

The authors declare no conflicts of interest.

## Data Availability

Data are available from the corresponding author on request.
